# Aquatic metagenomes implicate *Thaumarchaeota* in global cobalamin production

**DOI:** 10.1038/ismej.2014.142

**Published:** 2014-08-15

**Authors:** Andrew C Doxey, Daniel A Kurtz, Michael DJ Lynch, Laura A Sauder, Josh D Neufeld

**Affiliations:** 1 Department of Biology, University of Waterloo, Waterloo, Ontario, Canada

## Abstract

Cobalamin (vitamin B_12_) is a complex metabolite and essential cofactor required by many branches of life, including most eukaryotic phytoplankton. Algae and other cobalamin auxotrophs rely on environmental cobalamin supplied from a relatively small set of cobalamin-producing prokaryotic taxa. Although several *Bacteria* have been implicated in cobalamin biosynthesis and associated with algal symbiosis, the involvement of *Archaea* in cobalamin production is poorly understood, especially with respect to the *Thaumarchaeota*. Based on the detection of cobalamin synthesis genes in available thaumarchaeotal genomes, we hypothesized that *Thaumarchaeota*, which are ubiquitous and abundant in aquatic environments, have an important role in cobalamin biosynthesis within global aquatic ecosystems. To test this hypothesis, we examined cobalamin synthesis genes across sequenced thaumarchaeotal genomes and 430 metagenomes from a diverse range of marine, freshwater and hypersaline environments. Our analysis demonstrates that all available thaumarchaeotal genomes possess cobalamin synthesis genes, predominantly from the anaerobic pathway, suggesting widespread genetic capacity for cobalamin synthesis. Furthermore, although bacterial cobalamin genes dominated most surface marine metagenomes, thaumarchaeotal cobalamin genes dominated metagenomes from polar marine environments, increased with depth in marine water columns, and displayed seasonality, with increased winter abundance observed in time-series datasets (e.g., L4 surface water in the English Channel). Our results also suggest niche partitioning between thaumarchaeotal and cyanobacterial ribosomal and cobalamin synthesis genes across all metagenomic datasets analyzed. These results provide strong evidence for specific biogeographical distributions of thaumarchaeotal cobalamin genes, expanding our understanding of the global biogeochemical roles played by *Thaumarchaeota* in aquatic environments.

## Introduction

Cobalamin (vitamin B_12_) is an enzyme cofactor that many prokaryotic and eukaryotic species use to catalyze rearrangement-reduction or methyl transfer reactions involved primarily in amino acid synthesis (i.e., cobalamin-dependent methionine synthase), return of carbon to central metabolism via the tricarboxylic acid cycle (i.e., adenosylcobalamin-dependent methylmalonyl-CoA mutase) and synthesis of DNA (ribonucleotide reductase). Other cobalamin-dependent enzymes include ethanolamine ammonia-lyase, methylaspartate ammonia-lyase and methylaspartate mutase (reviewed in Sañudo-Wilhelmy *et al.* (2014)). Cobalamin is related to heme, chlorophyll and the F_420_ coenzyme of methanogens, and contains a tetrapyrrole corrin ring system surrounding a central cobalt atom. The fifth coordinated position of Co^3+^ is occupied by a dimethyl-benzamidazole nucleotide loop and the sixth catalytic upper ligand position is either occupied by a methyl group (i.e., methylcobalamin) or with a deoxyadenosine (i.e., adenosylcobalamin).

Because of the absolute requirement of cobalamin coenzymes by many marine bacterioplankton and most cultured phytoplankton, the supply of B vitamins (including vitamins B_1_, B_7_ and B_12_) have been implicated in controlling bloom distributions and species successions in the ocean water column (Sañudo-Wilhelmy *et al.,* 2006; Bertrand *et al.*, 2007; Gobler *et al.*, 2007). In particular, widespread nutritional auxotrophy results in a complete dependence by many marine organisms on an exogenous source of cobalamin. Auxotrophic phytoplankton are thought to establish symbiotic relationships with bacterial partners to obtain this vitamin (Croft *et al.*, 2005; Grant *et al.*, 2014), providing a source of organic carbon to the prokaryotic symbionts in return. Another strategy is to develop costly alternative pathways (e.g., cobalamin-independent methionine synthase) or to scavenge organic nutrients directly from the environment, which has been suggested for the ubiquitous *Pelagibacter ubique* of the SAR11 clade (Tripp *et al.*, 2008) and for a recently discovered class of uncultured marine *Euryarchaeota* (Iverson *et al.*, 2012).

Cobalamin biosynthesis represents one of the most complex and ancient metabolic pathways known (Raux *et al.*, 1999), and this vitamin has been described as ‘nature's most beautiful cofactor' (Stubbe, 1994). Owing to its elaborate structure, only a limited complement of *Bacteria* and *Archaea* are known to produce cobalamin. The ‘Black Queen Hypothesis' (i.e., in the card game ‘Hearts', players avoid holding the queen of spades; Morris *et al.*, 2012) has been invoked to explain why cobalamin producers maintain biosynthesis of cobalamin for potential community benefit, despite the high metabolic cost for those few taxa responsible for its production (Giovannoni, 2012). Based on genome analysis, known dominant marine bacterial taxa implicated in cobalamin production include select members of the *Cyanobacteria*, *Alphaproteobacteria*, *Gammaproteobacteria* and *Bacteroidetes* (Sañudo-Wilhelmy *et al.*, 2014). These characterized cobalamin producers synthesize cobalamin by either aerobic or anaerobic pathways that involve 20 enzymatic steps from a uroporphyrinogen III precursor, sharing several homologous enzymes, but with oxygen-requiring or oxygen-sensitive steps, depending on the pathway (Moore *et al.*, 2013). The aerobic pathway involves a late-insertion of the cobalt ion into the already contracted tetrapyrrole structure, whereas the early insertion pathway can operate under anoxic conditions and incorporates cobalt prior to ring contraction.

Although genomic analyses have implicated *Archaea* in cobalamin production (Rodionov *et al.*, 2003), examples have been limited to halophilic *Crenarchaeaota* of hypersaline ponds and marine methanogens from the *Euryarchaeota*. To our knowledge, no study has reported a potential cobalamin synthesis role for the *Thaumarchaeota*. This is surprising because *Thaumarchaeota* are widespread in aquatic and terrestrial environments (Francis *et al.*, 2005; Leininger *et al.*, 2006; Prosser and Nicol, 2008) and dominate marine water columns (Wuchter *et al.*, 2006). Known *Thaumarchaeota* possess autotrophic and oligotrophic metabolisms that are fueled by ammonia oxidation, and may be stimulated by organic molecules such as pyruvate (Tourna *et al.,* 2011; Stahl and de la Torre, 2012). Implicating cobalamin synthesis in thaumarchaeotal metabolism has important global biogeochemical and ecological implications, especially for the marine environment, where *Thaumarchaeota* are estimated to account for ∼20% of all prokaryotic cells (Karner *et al.*, 2001).

Based on our observation of cobalamin synthesis genes within available thaumarchaeotal genomes (Hallam *et al.*, 2006; Spang *et al.*, 2010; Walker *et al.*, 2010; Blainey *et al.*, 2011; Kim *et al.*, 2011; Lebedeva *et al.*, 2013; Lloyd *et al.*, 2013; Luo *et al.*, 2014), combined with the ubiquity and abundance of these organisms, we hypothesized that *Thaumarchaeota* represent numerically relevant marine cobalamin producers. We also predicted that cobalamin synthesis gene distributions would demonstrate biogeographical patterns, with correlations to measured physical and chemical characteristics. Leveraging available marine metagenomic libraries, we demonstrate depth-specific, latitudinal and temporal gradients of thaumarchaeotal cobalamin synthesis genes. We also observed niche partitioning of phylum-specific cobalamin synthesis, which reinforces that vitamin synthesis is a coordinated and costly keystone ecosystem function provided by individual, rather than collective, community members. Our results implicate *Thaumarchaeota* among dominant global cobalamin producers and add vitamin B_12_ production to the roles recognized for these abundant and enigmatic marine microbial community members.

## Materials and methods

### Genome pathway analysis

The Integrated Microbial Genomes system (Markowitz *et al.*, 2014) was used to detect matches to all enzymes within the Kyoto Encyclopedia of Genes and Genomes (KEGG; Kanehisa *et al.*, 2008) reference pathway map for ‘porphyrin and chlorophyll metabolism' (map00860), containing both the aerobic and anaerobic cobalamin biosynthesis pathways. The presence of each enzyme was assessed within all available archaeal genomes, and these data were retrieved to generate a presence/absence matrix. The proportion of archaeal genomes possessing each enzyme within the pathway was used to compute an archaeal ‘conservation score'. This score was then mapped onto a pathway heatmap using the KEGG Mapper tool (http://www.genome.jp/kegg/mapper.html).

### Metagenome datasets and processing

We retrieved 430 aquatic metagenomes from 28 studies from the Community Cyberinfrastructure for Advanced Microbial Ecology Research and Analysis (CAMERA) portal on 8 January 2014 (see Supplementary Table S1 for a full list of accession numbers). Included studies spanned all available marine and freshwater metagenomes, and excluded data from engineered environments or metagenomes related to host-associated, virus-, or bacteria-specific studies. Given that polar marine *Thaumarchaeota* were recently implicated in urea-fueled nitrification (Alonso-Sáez *et al.*, 2012), we also included the specific Arctic metagenome used in this previous study (NCBI SRA accession number ERP001178).

### Pipeline for detection and taxonomic classification of cobalamin biosynthesis genes

Metagenome profile Hidden Markov Models (profile-HMMs) were used for genes of the cobalamin biosynthesis pathway that were retrieved from the TIGRfam (Haft *et al.*, 2003) and Pfam (Bateman *et al.*, 2004) databases, as based on the KEGG pathway (map00860). A total of 11 genes (*cobA_cysG* C-terminal domain (TIGR01469), *cobI_cbiL* (TIGR01467), *cobJ_cbiH* (TIGR01466), *cobM_cbiF* (TIGR01465), *cbiT_cobL* (TIGR02469), *cbiE_cobL* (TIGR02467), *cbiC_cobH* (PF02570.10), *cbiA_cobB* (TIGR00379), *cbiB_cobD* (TIGR00380), *cbiP_cobQ* (TIGR00313), *cobS* (TIGR00317)) were selected as cobalamin pathway markers for further analysis due to their broad distribution throughout the cobalamin biosynthesis pathway. For each of these 11 genes, the corresponding HMM was used to scan for homologs in all sequence reads of each metagenome, which were initially processed into open reading frames using FragGeneScan (Rho *et al.*, 2010). The program hmmsearch within HMMER version 3.1b1 (hmmer.org) was used with default parameters and an *E*-value threshold of 1 × 10^−6^. The use of profile-HMMs based on protein family alignments eliminated bias that would have otherwise been introduced by homology searches based on single sequence queries. Tools and taxonomy indices from the Krona package (Ondov *et al.*, 2011) were used to assign taxonomy to recovered hits based on their top BLAST matches in the NCBI refseq database (version 60) with an *E*-value threshold of 1 × 10^−6^.

### Per-taxon cobalamin gene contribution

Taxonomic frequency profiles were generated by dividing the number of cobalamin synthesis hits per taxon by the total number of hits for a given gene category. Removing samples with low overall cobalamin synthesis gene frequencies (i.e., *n*<10) reduced potential statistical bias resulting from low sample sizes. To generate an overall measure of per-taxon cobalamin gene contributions for each sample, we combined data for the 11 cobalamin marker genes as follows. For a given dataset, *D*, the per-taxon cobalamin (*cob/cbi*) gene contribution (*D*_t_) was computed as:


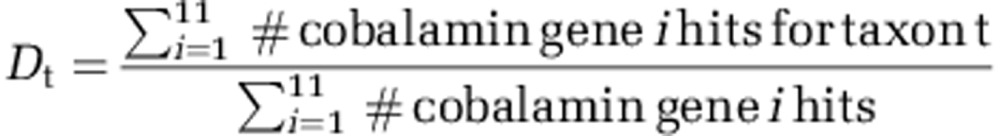


### Metadata, correlation and cluster analysis

Correlation analyses of numerical metadata from each metagenome compared individual physical and chemical characteristics with per-taxon cobalamin gene contributions (i.e., *D*_t_ from above). We analyzed metadata correlations across all individual metagenome datasets and also for pooled sample datasets for larger studies, with many samples characterized by common study-specific metadata. Standard Pearson correlations were used to calculate correlation coefficients (*r*). Maps were generated with the maps package in R (http://cran.r-project.org/web/packages/maps/index.html), with metagenome samples plotted by their associated latitude and longitude metadata. Hierarchical clustering was performed using the Ward method with Manhattan distances in R.

### Phylogenetic marker gene analysis

The full analysis described above was repeated using the Pfam profile-HMMs of three universally conserved phylogenetic marker genes (Ribosomal_S12_S23 (PF00164), Ribosomal_L18e (PF00828), and Ribosomal_S9 (PF00380)) that were selected from a previous publication (Lang *et al.*, 2013).

## Results

### Thaumarchaeotal cobalamin synthesis

**Figure 1:**
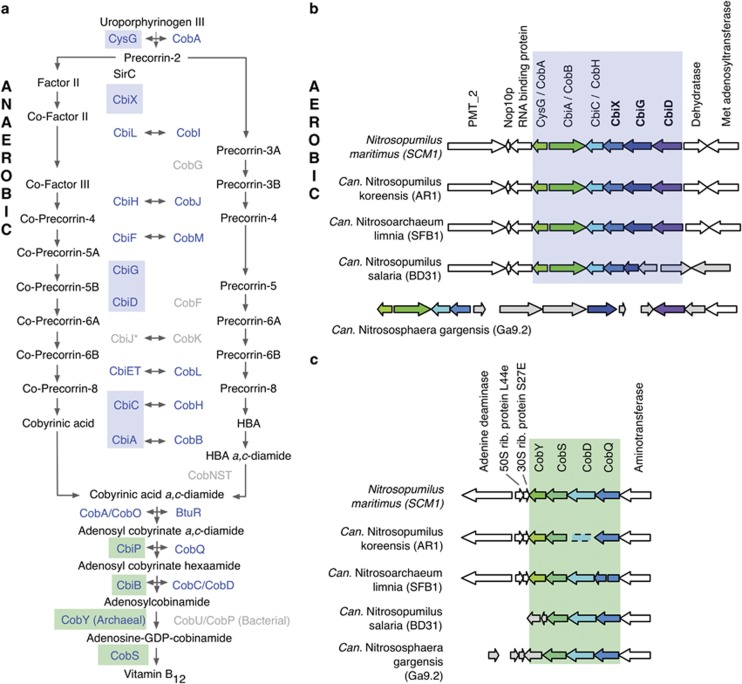
Identification of the cobalamin synthesis pathway and associated gene clusters in available thaumarchaeotal genomes. The pathway in (**a**) is adapted from the studies by Moore *et al.* (2013) and Raux *et al.* (1999). Horizontal arrows indicate homology between aerobic and anaerobic pathway enzymes. Grey enzyme names were not detected in thaumarchaeotal genomes. *CbiJ is also not present in other known archaeal cobalamin producers and therefore should not be considered an essential gene for this pathway in *Thaumarchaeota*. (**b**) A six-member cobalamin synthesis gene cluster with conserved synteny across several thaumarchaeotal genomes. The cluster encodes enzymes (highlighted by blue boxes in (**a**)) in the upper pathway, and includes three enzymes (bolded) specific to the anaerobic pathway. (**c**) A four-member gene cluster possessing enzymes (highlighted by green boxes in (**a**)) corresponding to the final steps of cobalamin synthesis. The apparent missing *cobD* gene in AR1 may be a genome annotation error given that it appears to be in the genome but may be truncated (data not shown).

Genomic analysis of the ammonia-oxidizing archaeon *Nitrosopumilus maritimus* SCM1 and related thaumarchaeotal representatives indicates a genetic capacity for cobalamin biosynthesis. Using *N. maritimus* SCM1 as a reference, we identified a complete biosynthetic pathway and separate gene clusters encoding distinct stages of cobalamin biosynthesis (Figure 1, Supplementary Figures S1 and S2). Although both the aerobic and anaerobic pathways for cobalamin synthesis share a majority of their enzymes, several steps and associated enzymes are specific to each (Figure 1a). *N. maritimus* and related *Thaumarchaeota* (Figures 1b and c) lack genes encoding enzymes of the aerobic pathway (*CobG*, *CobF*, *CobNST*) and possess genes encoding enzymes specific to the anaerobic biosynthesis pathway (*CbiX*, *CbiG*, *CbiD*). This is supported by the presence and absence of marker genes as defined by the KEGG (Supplementary Figures S1 and S2). *N. maritimus* and other *Thaumarchaeota* were also found to encode cobalamin-dependent enzymes such as methionine synthase (Nmar_1267), ribonucleotide reductase (Nmar_1627), and methylmalonyl CoA mutase (B_12_ binding domain, Nmar_0958), as well as probable cobalt transporters (Nmar_0878).

As further support for cobalamin biosynthetic potential, we identified dual *cob/cbi* gene clusters conserved across all known ammonia-oxidizing thaumarchaeotal genomes. The two relevant gene clusters are present in the genomes of *Candidatus* Nitrosoarchaeum limnia, *Candidatus* Nitrosopumilus salaria, *Candidatus* Nitrososphaera gargensis, and *Candidatus* Nitrosopumilus koreensis AR1 (Figures 1b and c), in addition to *Cenarchaeum symbiosum* and *Candidatus* Nitrosotenuis uzonensis (Supplementary Figure S2 and data not shown, respectively). With the possible exception of *Can*. N. gargensis, these gene clusters exhibit highly conserved synteny (Figures 1b and c, Supplementary Figure S2), indicative of selection for coordinated regulation. Furthermore, these gene clusters encode separate stages of cobalamin biosynthesis, with the first stage encoding six enzymes for the early pathway converting uroporphyrinogen III to cobyrinic acid a,c-diamide (Figures 1a and b) and the second stage encoding four enzymes for the later conversion of cobyrinic acid a,c-diamide to vitamin B_12_ (Figures 1a and c).

Our results also show that many *Archaea* possess a similar complement of cobalamin biosynthetic genes, which cluster together and co-occur genomically (Supplementary Figures S2 and S3). This, along with the observed sequence and gene order conservation, suggests widespread functional conservation of the cobalamin biosynthetic pathway among the *Archaea*.

### Environmental distributions of cobalamin synthesis genes

We searched for cobalamin biosynthesis genes within 430 metagenomes derived from 28 studies, representing a diverse range of marine, freshwater and hypersaline environments. Eleven cobalamin synthesis genes were selected based on their representation of both the aerobic and anaerobic pathway (Figure 1a), broad distribution throughout the cobalamin pathway and consistency with a core majority of genes used previously to identify cobalamin producers (Sañudo-Wilhelmy *et al.*, 2014).

**Figure 2:**
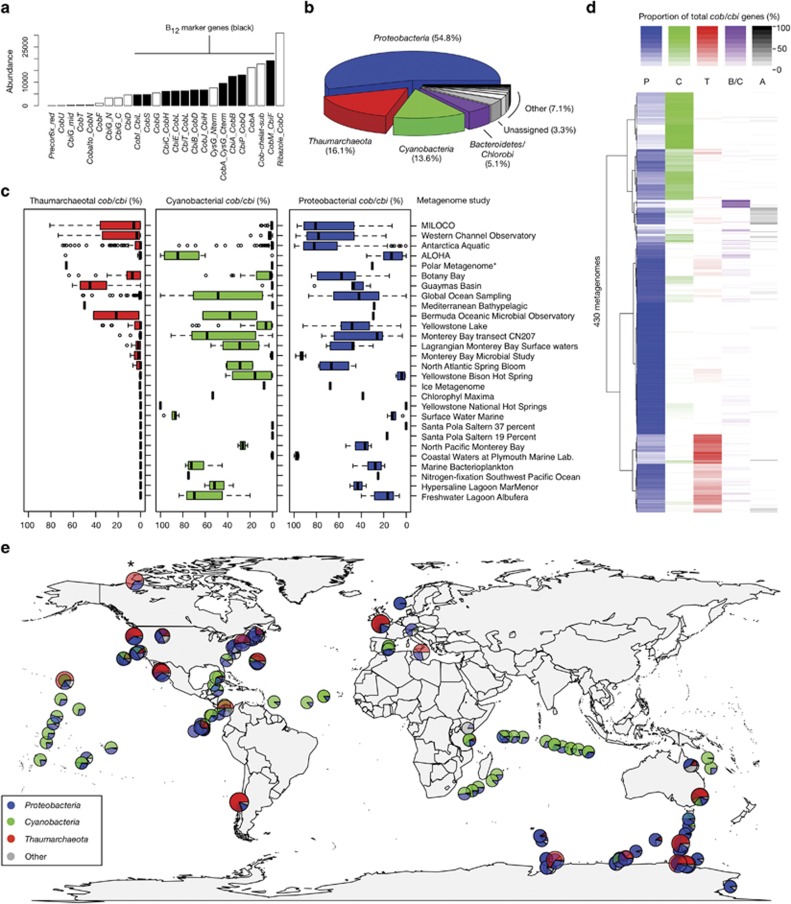
Survey of cobalamin synthesis genes across aquatic metagenomes. (**a**) The full set of *cob/cbi* genes and subset of eleven representative marker genes (black) used in the screen and their abundance (total number of detected genes). (**b**) Taxonomic breakdown of all detected *cob/cbi* genes across 430 aquatic metagenomes. (**c**) Distributions of thaumarchaeotal, cyanobacterial and proteobacterial cobalamin gene contributions (% of total *cob/cbi* genes) for 28 different studies. (**d**) Clustering and heatmap visualization of taxonomic composition of *cob/cbi* genes for the 430 metagenomes. The color scale reflects the proportion of *cob/cbi* genes in each sample affiliated with a particular taxonomic group. More information on each sample can be found in Supplementary Table S1. (**e**) Global distribution of cobalamin synthesis genes. To emphasize *Thaumarchaeota*-rich metagenomes, data points with higher thaumarchaeotal contributions have been slightly enlarged and overlayed preferentially in cases where there are overlapping data points. The *Thaumarchaeota*-rich Arctic metagenome described in the text is indicated by an asterisk.

Our screen identified 187 223 total cobalamin biosynthetic genes across all metagenomes, 95 911 of which matched the 11 marker genes (Figure 2a). Taxonomic assignments classified these 95 911 genes into three main phyla (Figure 2b): *Proteobacteria* (P), *Cyanobacteria* (C), and *Thaumarchaeota* (T). At the species level, *Thaumarchaeota* dominated among prevalent organisms (Table 1), comprising three of the four most frequent contributors of *cob/cbi* genes overall. *Thaumarchaeota* were also significantly overrepresented within the context of cobalamin synthesis (contributing 16.1% of *cob/cbi* genes) relative to their community abundance (contributing 2.5% of ribosomal marker genes). These results indicate that *Thaumarchaeota* are abundant global cobalamin producers through the anaerobic biosynthetic pathway, and may be a keystone species for cobalamin production.

In addition to evaluating all metagenome datasets together, we partitioned studies into their individual metagenome samples (430 in total), each of which was associated with distinct environmental parameters. Environment-specific metagenomes varied widely in taxonomic composition of cobalamin genes, within and between studies (Figure 2c). Hierarchical clustering of cobalamin taxonomic profiles of all metagenomes demonstrated visual ecotype partitioning (Figure 2d), including distinct T, P and C environments, or mixed environments (e.g., P+C or P+T). These recurring ecotypes show broad geographic distribution and non-uniform latitudinal gradients (Figure 2e).

**Figure 3:**
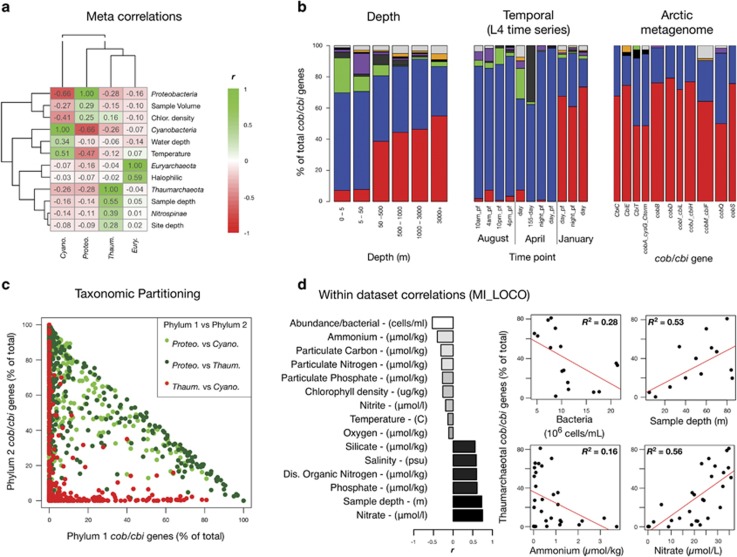
Phylum and metadata correlations. (**a**) Correlation matrix for all species-metadata correlations with *r*>0.25. Correlations were measured between numerical metadata from all 430 metagenome samples and the per-taxon proportion of cobalamin genes. Correlations between taxonomic proportions were also measured to identify potential species–species associations. (**b**) Left: proportion of cobalamin synthesis genes vs depth for three phyla (computed using all 430 metagenome samples). Middle: proportion of cobalamin synthesis genes vs different time points from the 2008–2009 L4 station time series. Right: per-gene taxonomic proportions of cobalamin genes for an Arctic metagenome. *Thaumarchaeota* (red), *Cyanobacteria* (green), *Proteobacteria* (blue), Unclassified (dark grey), *Bacteroidetes/Chlorobi* (purple), *Actinobacteria* (black), *Firmicutes* (orange), and Other (light gray). Bar widths are proportional to the square root of the sample sizes. (**c**) Phyla-vs-Phyla contributions of cobalamin synthesis genes across all 430 metagenome samples. All correlations exhibit a negative linear relationship, except for *Thaumarchaeota*-vs-*Cyanobacteria*, which exhibits an either-or relationship, suggestive of competitive or niche exclusion. (**d**) Correlation analysis for the MI_LOCO metagenome. The strongest correlating variables with thaumarchaeotal *cob/cbi* gene contribution are shown on the left, and plots for the top two positively (nitrate concentration and sample depth) and negatively correlated (ammonium and bacterial cell concentration) variables are shown on the right.

*Thaumarchaeota* ranged from undetected to contributing as much as ∼70–80% of the cobalamin genes in various samples from the Microbial Initiative in Low Oxygen areas off Concepcion and Oregon (MI_LOCO; http://mi-loco.coas.oregonstate.edu), Western Channel Observatory Microbial Metagenomic Study (http://camera.crbs.ucsd.edu/projects/details.php?id=CAM_PROJ_WesternChannelOMM; Gilbert *et al.*, 2010) and Antarctica metagenome (http://camera.crbs.ucsd.edu/projects/details.php?id=CAM_PROJ_AntarcticaAquatic) studies (Figure 2c, Supplementary Table S1). In addition, *Thaumarchaeota* were the dominant source of *cob/cbi* genes in several metagenomes included in this analysis (Supplementary Table S1), including the Arctic metagenome (66%, Figure 3b), deep-ocean bathypelagic habitat metagenome (50%), the station ALOHA (500 m depth) metagenome (67%) and individual sites from the Global Ocean Surveys dataset. Several metagenome studies included few samples (e.g., a single sample for the Arctic metagenome), and may therefore not be representative of their respective environments.

### Thaumarchaeotal cobalamin genes correlate with environmental variables

The observed variation (Figure 2c) and clustering (Figures 2d and e) of distinct and recurring cobalamin ecotypes (Figures 2d and e) indicate that specific environmental variables drive the dynamics of cobalamin production. We performed correlation analyses across and within all metagenome datasets, and identified specific factors, including depth, nutrient levels, temperature and seasonality, that correlate with the taxonomic composition of cobalamin synthesis genes (Figure 3). The proportion of thaumarchaeotal cobalamin genes correlated most strongly (*r*=0.55) with sample depth (Figures 3a and b), consistent with observations of thaumarchaeotal dominance in deep water column metagenome studies (Figure 3 and Supplementary Figure S4). The correlation with depth was stronger across 63 samples from the MI_LOCO study (*r*=0.73, Figure 3d), which is likely attributed to higher within-study methodological consistency.

Further analysis of the MI_LOCO dataset revealed additional correlating variables that are consistent with known aspects of thaumarchaeotal ecology. Levels of thaumarchaeotal cobalamin synthesis genes exhibited strong positive correlations with numerous factors (Figure 3d), including nitrate (*r*=0.75), sample depth (*r*=0.73), phosphate (*r*=0.61) and dissolved organic nitrogen (*r*=0.60). The strongest negative correlations with thaumarchaeotal *cob/cbi* genes included bacterial cell number (*r*=−0.53) and ammonium concentration (*r*=−0.40). Low ammonium mixing ratios are consistent with a role for marine *Thaumarchaeota* as high affinity ammonia oxidizers (Martens-Habbena *et al.*, 2009; Horak *et al.*, 2013; Nakagawa and Stahl, 2013).

Taxonomic cobalamin gene contributions also varied temporally. This was most apparent in a single metagenomic study from the Western English Channel time-series experiments sampled from station L4 (Figure 3b). The average proportion of thaumarchaeotal cobalamin synthesis genes was low (∼3%) for the August and April time points, but increased up to 73% in January, suggesting that *Thaumarchaeota* are dominant contributors to cobalamin production in cold or winter-associated marine waters.

Additional phyla-vs-phyla correlations revealed a strong anticorrelation between thaumarchaeotal and cyanobacterial cobalamin synthesis genes (Figure 3a and see either-or effect in Figure 3c). This was evident in additional analyses that revealed no apparent T+C-rich ecotypes (Figure 2d). Similar patterns were observed for three phylogenetic marker genes (Supplementary Figure S5), suggesting a broader niche partitioning or exclusion between these two major marine clades. Unlike *Thaumarchaeota*, cyanobacterial cobalamin synthesis gene abundances correlated positively with temperature (Figure 3a), consistent with warm water cyanobacterial blooms, and negatively with depth (Figure 3a). We also identified a positive correlation between cobalamin gene proportions of *Thaumarchaeota* and *Nitrospinae,* a nitrite-oxidizing bacterial phylum. This likely reflects a functional association between these two groups, linked by nitrification.

## Discussion

Available genomes and metagenomic studies provide strong support that *Thaumarchaeota* are globally important vitamin B_12_ producers. Although cobalamin synthesis is distributed throughout both *Archaea* and *Bacteria*, only a restricted subset of microorganisms encodes this genetic capacity. Bacterial cobalamin synthesis is associated with select members of *Cyanobacteria*, *Alphaproteobacteria*, *Gammaproteobacteria*, and *Bacteroidetes* (Sañudo-Wilhelmy *et al.*, 2014), which are consistent with the dominant phyla observed in the metagenomic datasets analyzed here (Figure 2b). Within the *Archaea*, only methanogenic *Euryarchaeota* and extremophilic crenarchaeal representatives were recognized as cobalamin producers prior to this study. For example, methanogens are known to produce and require cobalamin for acetoclastic and hydrogenotrophic methanogenesis (reviewed in DiMarco *et al.* (1990)). In addition, cobamides are produced and required by *Halobacterium* (Woodson *et al.*, 2003) and known sulfur-metabolizing *Crenarchaeaota* (Kräutler *et al.*, 1988). Given that *Thaumarchaeaota* are estimated to represent the most abundant *Archaea* on the planet, and are among the most abundant prokaryotes in the ocean (Karner *et al.*, 2001), the capacity to synthesize vitamin cofactors within this phylum implies global biogeochemical, metabolic and ecological significance.

Genetic capacity for cobalamin synthesis is very tightly conserved. For example, several genes from the multi-step cobalamin synthesis pathway are considered reliable indicators for the entire pathway. If a genome encodes *cbiA/cobB*, *cbiC/cobH* or *cobT*, then the entire cobalamin synthesis pathway is likely encoded and expressed in that microorganism (Bertrand *et al.*, 2011). Indeed, all available thaumarchaeotal genomes encode this capacity, and demonstrate conserved clusters of cobalamin synthesis genes (Figure 1 and Supplementary Figures S2 and S3). Providing physiological support for cobalamin synthesis by the *Thaumarchaeota*, existing pure cultures of ammonia-oxidizing archaea (including, *N. maritimus* SCM1 and *Nitrosospaera viennensis*) are grown in medium prepared without vitamin supplementation (Martens-Habbena and Stahl, 2011; Tourna *et al.,* 2011). Given that the gene for the low efficiency B_12_-independent methionine synthesis pathway (*metE*) is absent in the genome of *N. maritimus* SCM1, growth in the absence of vitamin supplementation provides strong supporting evidence for thaumarchaeotal cobalamin synthesis. Direct measurements of gene expression and cobalamin production by pure cultures of *N. maritimus* SCM1 and additional *Thaumarchaeota* would confirm thaumarchaeotal cobalamin synthesis and provide additional information on the structure, regulation and fate of this vitamin when produced by marine *Thaumarchaeota*. Although all known ammonia-oxidizing archaea possess the genetic capacity for cobalamin synthesis, the absence of homologous genes in *Caldiarchaeaum subterranean* (Supplementary Figure S2), a deep branching archaeon related to the *Thaumarchaeota* (Nunoura *et al.*, 2011), suggests that cobalamin synthesis is not universally distributed among related *Archaea*.

Cobalamin gene analysis of available aquatic metagenomic datasets provides compelling support for niche specificity and biogeochemical controls on cobalamin production. Taxonomic affiliations indicate that although proteobacterial cobalamin synthesis genes can co-occur with either *Cyanobacteria* or *Thaumarchaeota*, these two non-proteobacterial cobalamin producers display strong mutual exclusion (Figures 2 and 3). This trend was also observed when repeating the analysis using ribosomal phylogenetic markers (Supplementary Figure S5), suggesting broad niche partitioning between these major marine groups. In addition to light sensitivity of *Thaumarchaeota* (described below), a possibility is that high cyanobacterial photosynthetic production of molecular oxygen interferes with the anaerobic thaumarchaeotal cobalamin biosynthetic pathway, which possesses an oxygen-sensitive intermediate (cobalt-precorrin-5B; Moore *et al.*, 2013). In addition to niche partitioning of thaumarchaeotal and cyanobacterial cobalamin synthesis genes, metadata correlations revealed a positive correlation with marine nitrite-oxidizing *Nitrospinae*, which is consistent with the an ammonia-oxidizing role for most marine *Thaumarchaeota*. *Nitrospina*-like bacteria have also been implicated as the primary nitrite oxidizers in oxygen minimum upwelling zones (Levipan *et al.*, 2014), which is consistent with high thaumarchaeotal abundance in these marine environments (Supplementary Figure S4).

The metagenomic data are also consistent with observed geographical and temporal patterns of thaumarchaeotal abundances. In particular, the urea-fueled polar and halocline abundances of *Thaumarchaeota* (Alonso-Sáez *et al.*, 2012) were reflected here in polar cobalamin synthesis gene abundances in the same datasets. Winter peak abundances of *Thaumarchaeota* (Wuchter *et al.*, 2006; Herfort *et al.*, 2007; Galand *et al.*, 2010; Pitcher *et al.*, 2011) are consistent with our observation of a winter thaumarchaeotal cobalamin gene maximum at station L4. In addition, previous research has reported high thaumarchaeotal abundance in oxygen minimum zones (Pitcher *et al.*, 2011; Peng *et al.*, 2013), which reflects our observation of abundant thaumarchaeotal cobalamin genes in the MI_LOCO metagenomes. The longstanding observation of abundant *Thaumarchaeota* (then ‘mesophilic *Crenarchaeota*') in deep marine water columns (Karner *et al.*, 2001; Sintes *et al.*, 2013) is also supported strongly by the metagenomic data survey conducted in this study. Importantly, polar environments, aphotic water columns, oxygen minimum zones, and winter time points share low light conditions, which would be a likely explanation for the observed anticorrelation between thaumarchaeotal and cyanobacterial cobalamin synthesis and ribosomal genes. The biogeographical cobalamin synthesis gene distributions observed in this study are consistent with observations of light-dependent decreases in marine thaumarchaeotal abundance below sea ice (Alonso-Sáez *et al.*, 2012) and known photoinhibition of thaumarchaeotal ammonia oxidation (Merbt *et al.*, 2012). Other factors proposed to explain aquatic thaumarchaeotal distributions include nutrient availability, temperature, dissolved oxygen and organic substrates (Peng *et al.*, 2013).

Observations of vitamin B_12_ maxima in ocean waters below the marine photic zone are consistent with an important role for *Thaumarchaeota* in marine cobalamin production, given their high proportional abundance with depth. Previous observations of increased cobalamin concentrations in ocean mesopelagic zones (Sañudo-Wilhelmy *et al.,* 2012) have led to suggestions that vitamin-producing microbial plankton below the photic zone are important contributors to marine biogeochemistry, in addition to essential suppliers of growth factors for phytoplankton and microbial communities in the photic zone (Sañudo-Wilhelmy *et al.*, 2014). Although ocean upwelling zones might be considered a mechanism for supplying cobalamin to surface waters, vitamin B_12_ concentrations did not correlate with upwelling index off the coast of Baja California, Mexico (Panzeca *et al.*, 2009). With respect to high latitude environments, cobalt supplementation of North Atlantic marine samples led to increased vitamin B_12_ production (Panzeca *et al.*, 2008). Our metagenomic analysis of high latitude samples implies that *Thaumarchaeota* were responsible for vitamin B_12_ production in this experiment, and implies that thaumarchaeotal productivity may be limited by cobalt concentrations. Importantly, vitamin B_12_ was shown to influence phytoplankton growth and community composition in samples taken from the subarctic Gulf of Alaska (Koch *et al.*, 2011), which is consistent with controls on phytoplankton growth by vitamin B_12_ in freshwater environments as well (Daisley, 1969; Cavari and Grossowicz, 1977). Together, these results provide early evidence that cobalamin production by *Thaumarchaeota* may directly influence phytoplanktonic communities in polar surface waters, freshwater and possibly in marine surface waters via mixing with ocean water column vitamin pools.

## Conclusion

Thaumarchaeotal vitamin production in most of the global ocean would have enormous potential impacts on microbial communities and food web dynamics. Here we provide evidence that *Thaumarchaeota* are among the most abundant global cobalamin vitamin producers. All known ammonia-oxidizing members of the *Thaumarchaeota* possess the genetic capacity to produce cobalamin, and data from multiple metagenomic studies reveal niche partitioning of this keystone community function. Thaumarchaeotal cobalamin synthesis genes demonstrate spatial and temporal biogeographical distributions. Combining pure culture experimental work and field studies will help assess the proportion of marine cobalamin pools derived from archaeal synthesis, identify the role of archaeal cobalamin in marine biogeochemistry, and characterize links between global thaumarchaeotal cobalamin production and microbial community composition and succession.

## Figures and Tables

**Table 1:** The 15 most abundant species contributing detected metagenomic *cob/cbi* genes across all datasets

*Species*	*Phylum/class*	*cob/cbi genes*
*Rhodobacterales* bacterium HTCC2255	*Alphaproteobacteria*	9091
***Candidatus* Nitrosopumilus sp. AR2**	*Thaumarchaeota*	4394
***Candidatus*** **Nitrosopumilus salaria**	*Thaumarchaeota*	4110
***Candidatus*** **Nitrosoarchaeum limnia**	*Thaumarchaeota*	3656
*SAR324 cluster bacterium* SCGC AAA001 C10	*Alphaproteobacteria*	3283
***Nitrosopumilus maritimus*** **SCM1**	*Thaumarchaeota*	2672
*Prochlorococcus marinus* str. MIT 9301	*Cyanobacteria*	2422
*Prochlorococcus marinus* str. AS9601	*Cyanobacteria*	2371
*Gamma proteobacterium* SCGC AAA007 O20	*Gammaproteobacteria*	2279
***Candidatus* Nitrosopumilus koreensis AR1**	*Thaumarchaeota*	2039
*Chlorobium phaeovibrioides* DSM 265	*Chlorobi*	1942
***Candidatus*** **Nitrosoarchaeum koreensis** **MY1**	*Thaumarchaeota*	1912
*Neptuniibacter caesariensis*	*Gammaproteobacteria*	1663
Endosymbiont of *Bathymodiolus* sp.	*Gammaproteobacteria*	1571
*Prochlorococcus marinus* subsp. *pastoris* str. CCMP1986	*Cyanobacteria*	1226

Taxonomic annotations were assigned based on the top BLAST match and are therefore dependent on the existing species diversity of the reference (NCBI) database. Thaumarchaeotal species are in bold.
