# Supplementary material for: Aquatic metagenomes implicate Thaumarchaeota in global cobalamin production
Source: ISME J. 2014 Aug 15;9(2):461–71. doi: 10.1038/ismej.2014.142 (PMC4303638; doi:10.1038/ismej.2014.142)
Supplement: Supplementary Information [file ismej2014142x1.doc]

**SUPPLEMENTARY INFORMATION**

**Aquatic metagenomes implicate *Thaumarchaeota* in global cobalamin production**

Andrew C. Doxey, Daniel A. Kurtz, Michael D. J. Lynch, Laura A. Sauder, Josh D. Neufeld

Department of Biology, University of Waterloo, Waterloo, Ontario, N2L 3G1, Canada

Corresponding authors:

Doxey AC, Department of Biology, University of Waterloo, 200 University Ave. West, Waterloo, Ontario, N2L 3G1, Canada

Neufeld JD, Department of Biology, University of Waterloo, 200 University Ave. West, Waterloo, Ontario, N2L 3G1, Canada

Running title: *Thaumarchaeota* are global cobalamin producers

Subject Category: Microbial ecology and functional diversity of natural habitats

Figure S1. Presence of the cobalamin biosynthesis pathway in *Nitrosopumilus maritimus* SCM1*.* Pathway information was obtained from the KEGG database (<http://www.genome.jp/kegg/>, pathway map nmr00860).

Figure S2. Presence/absence of cobalamin biosynthesis genes in 167 archaeal genomes (JGI data source, finished sequencing status as of April 13, 2014). Data were generated using KEGG pathway markers as implemented by the JGI Integrated Microbial Genomes (IMG) resource. All genes within the broader KEGG category of “Porphyrin and Chlorophyll Metabolism” were included. Key cobalamin synthesis genes have been labelled below the plot. Anaerobic marker genes (blue) are present in a majority of archaeal genomes, whereas aerobic marker genes (grey) are absent. The five reference thaumarchaeotal species are outlined by the upper box (“Thaum”), and the lower box indicates the species *Candidatus* Caldiarchaeum subterraneum, a deeply branching relative of the *Thaumarchaeota* that lacks cobalamin pathway genes.

Figure S3. Conservation of cobalamin biosynthesis genes across archaeal genomes. Each pathway step is coloured according to archaeal conservation (white, no conservation; red, maximum conservation) as defined by fractional occurrence (based on Figure S2). Blue steps indicate enzymes that were not included in the KEGG pathway analysis module.

Figure S4. Cobalamin taxonomic profiles for 14 selected metagenomic studies, with all individual study samples pooled. For each metagenome study, taxonomic proportions are shown for all nine cobalamin marker genes. *Thaumarchaeota* (red), *Cyanobacteria* (green), *Proteobacteria* (blue), Other (white). Bar widths are proportional to the square root of the sample sizes.

Figure S5. Phyla-vs-Phyla contributions of ribosomal genes across all 430 metagenome samples for A) Ribosomal_S12_S23 [PF00164], B) Ribosomal_L18e [PF00828], C) Ribosomal_S9 [PF00380], and D) an averaged analysis of these three ribosomal genes. Proteo. is *Proteobacteria*, Cyano. is *Cyanobacteria*, and Thaum. is *Thaumarchaeota*.

Table S1. List of metagenomes used for this study.
