# Supplementary figures and images for: Aquatic metagenomes implicate Thaumarchaeota in global cobalamin production
Source: ISME J. 2014 Aug 15;9(2):461–71. doi: 10.1038/ismej.2014.142 (PMC4303638; doi:10.1038/ismej.2014.142)

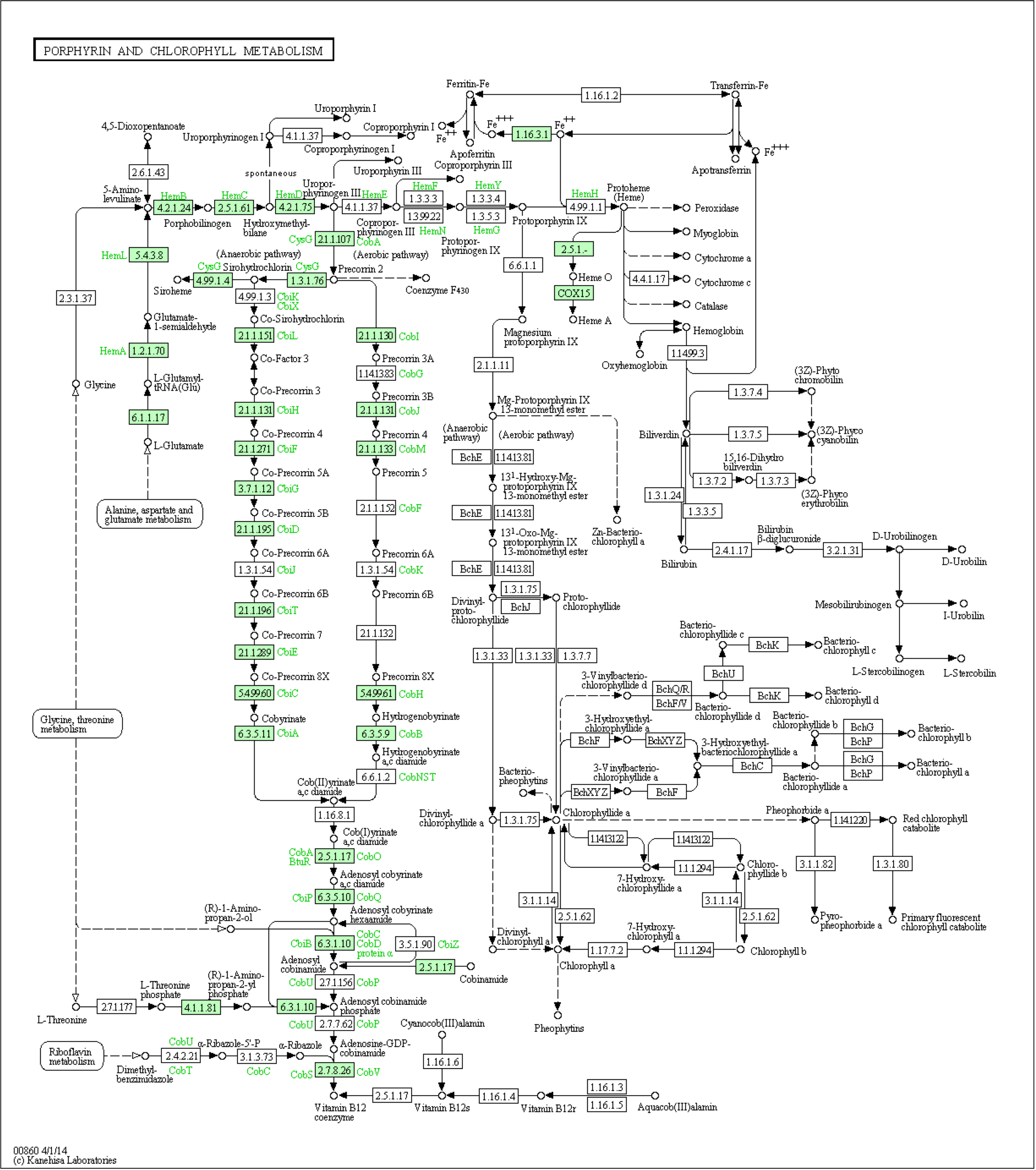

Supplement: Supplementary Figure 1 [file ismej2014142x2.tif]

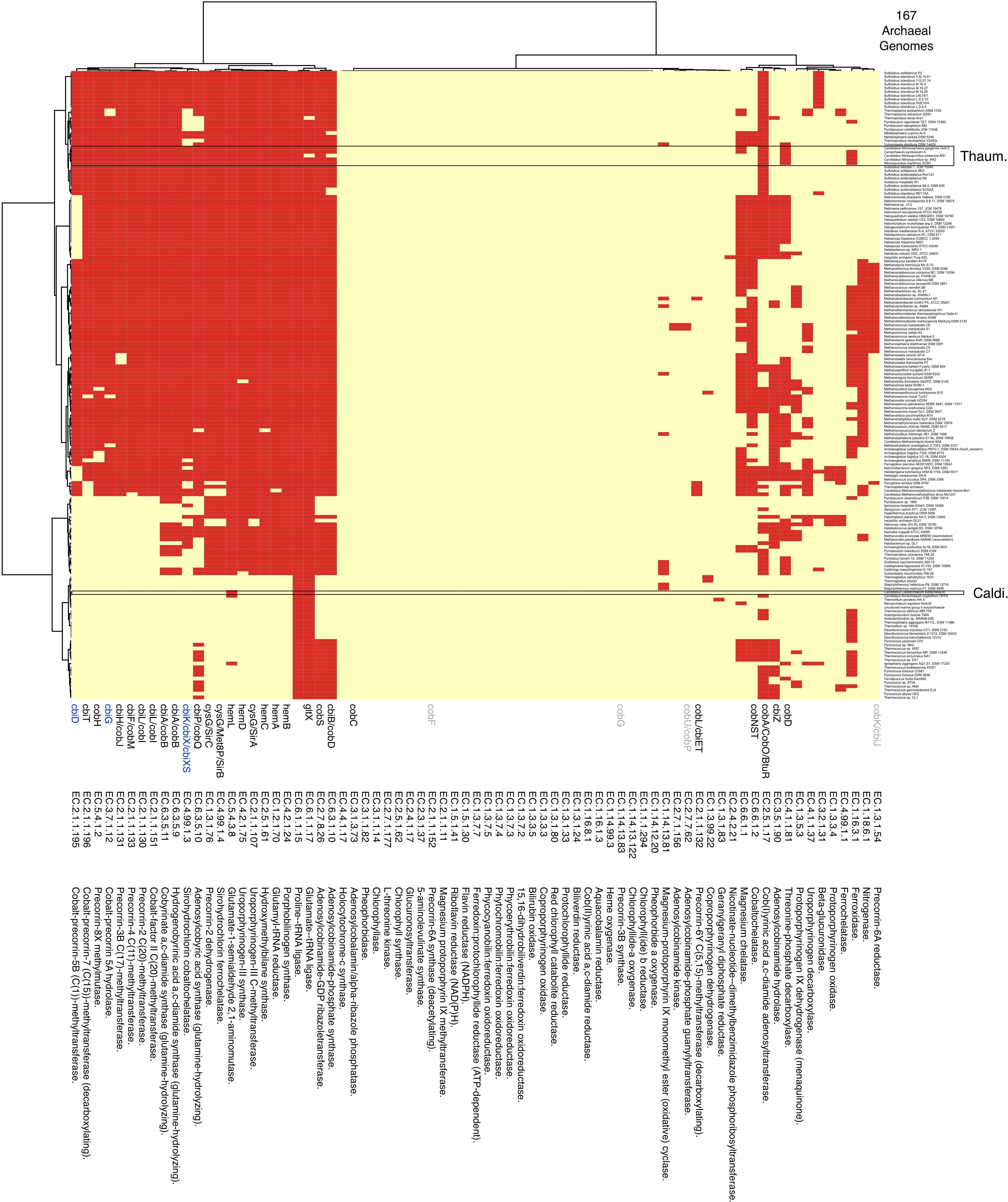

Supplement: Supplementary Figure 2 [file ismej2014142x3.tif]

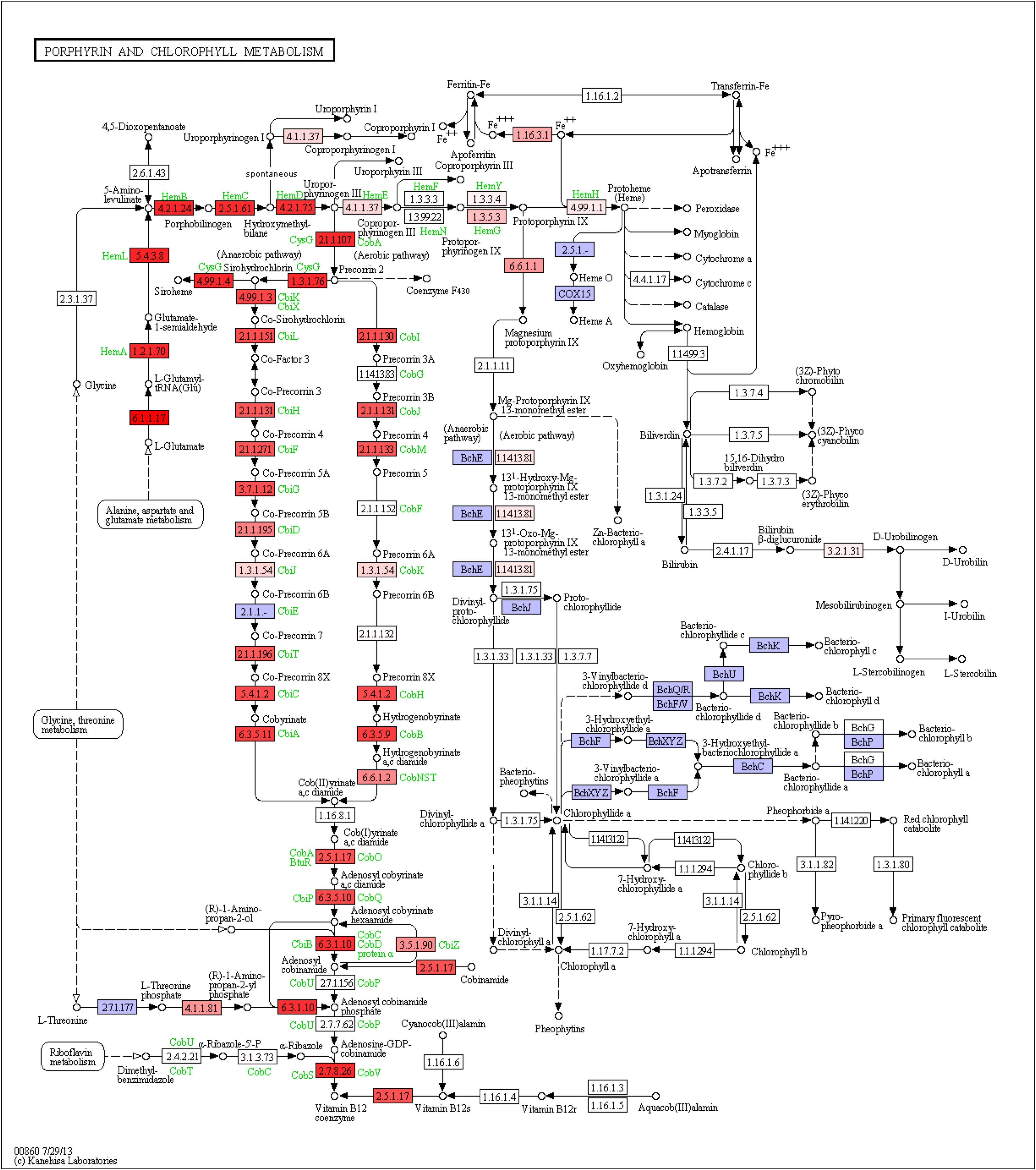

Supplement: Supplementary Figure 3 [file ismej2014142x4.tif]

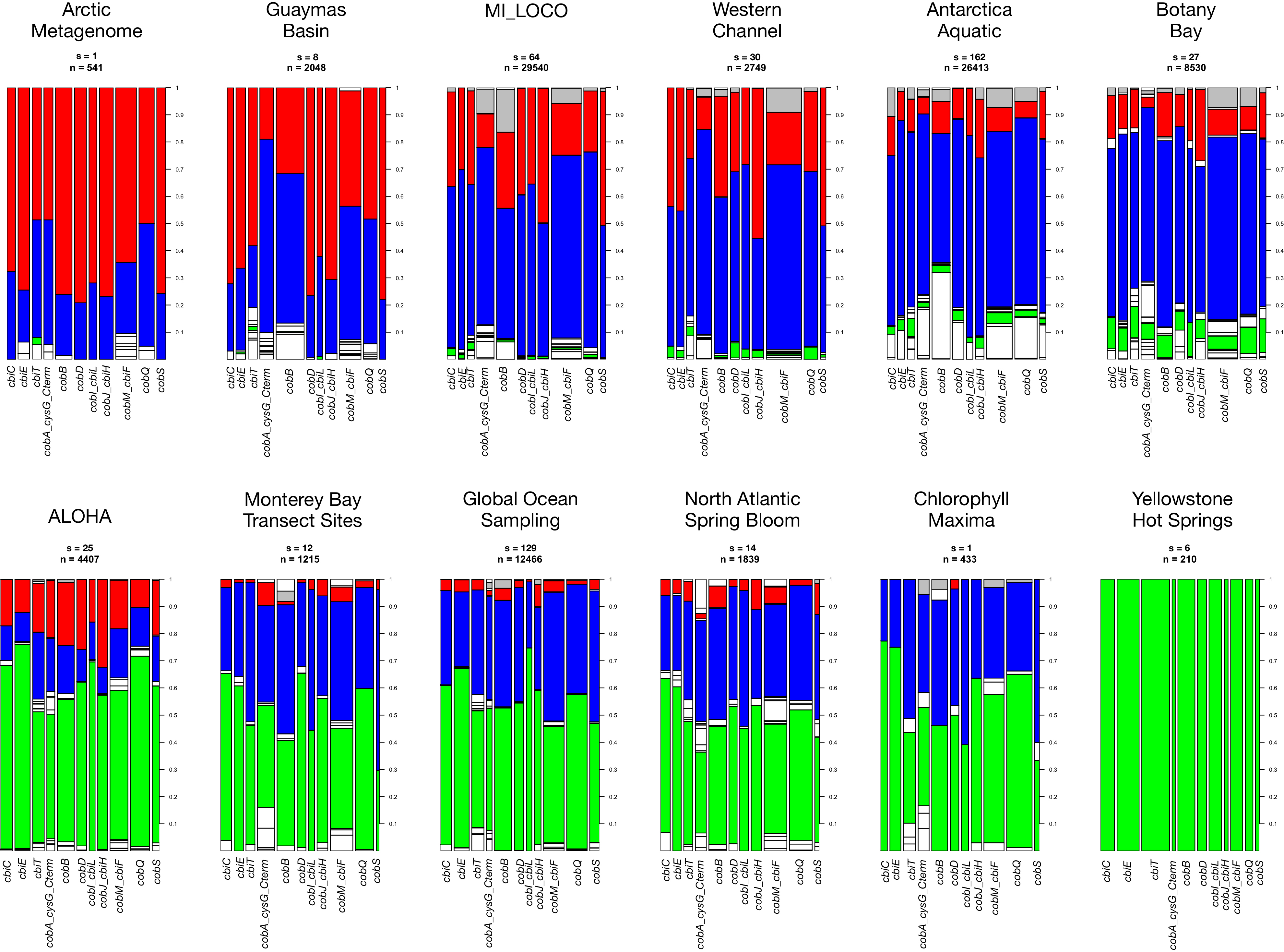

Supplement: Supplementary Figure 4 [file ismej2014142x5.tif]

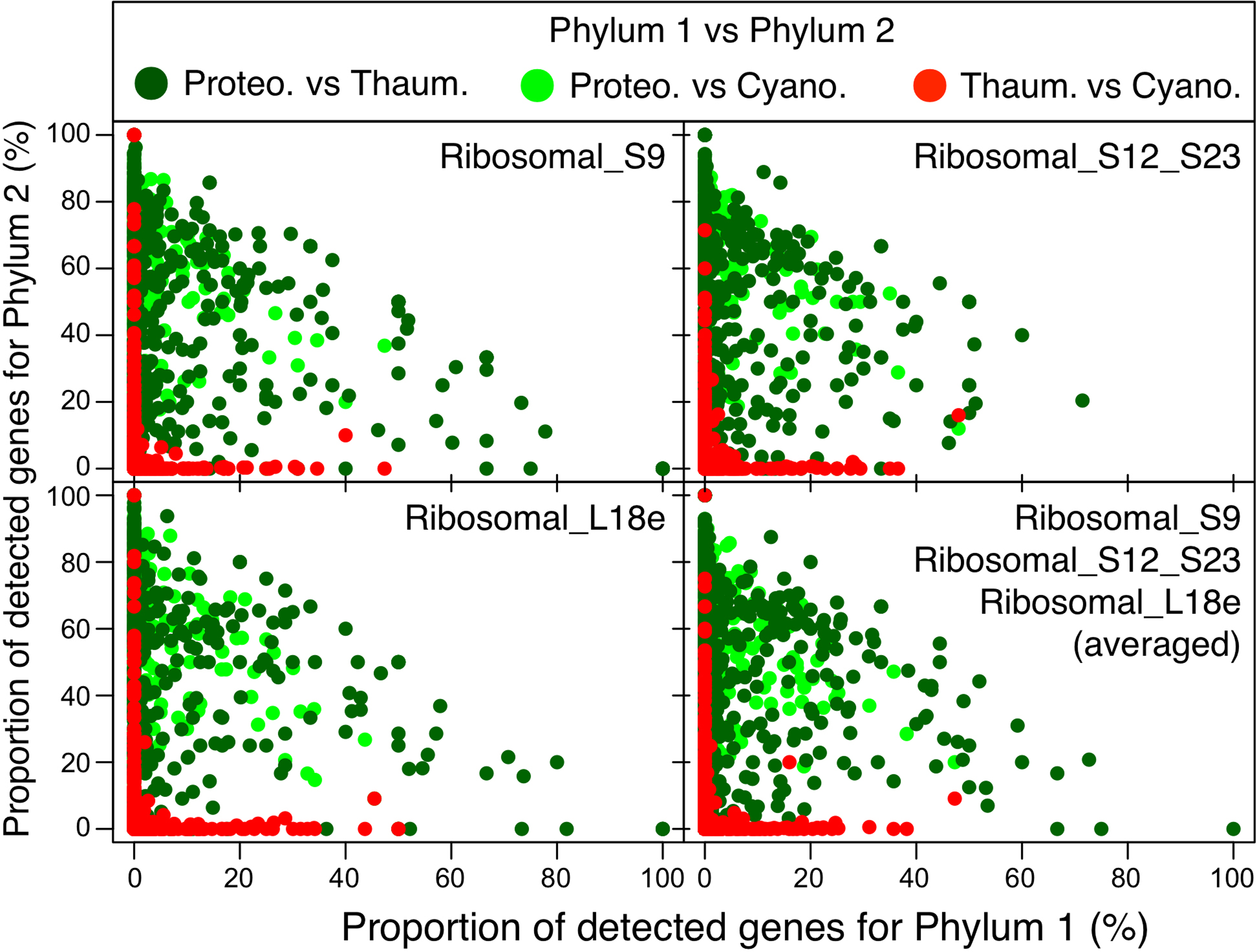

Supplement: Supplementary Figure 5 [file ismej2014142x6.tif]
